# Outcome and management in neonates with gastroschisis in the third millennium—a single-centre observational study

**DOI:** 10.1007/s00431-022-04416-9

**Published:** 2022-02-28

**Authors:** Lotta Räsänen, Helene Engstrand Lilja

**Affiliations:** 1 Department of Women’s and Children’s Health, Uppsala University, Uppsala, Sweden; 2 Section of Pediatric Surgery, Uppsala University Children’s Hospital, 751 85 Uppsala, Sweden

**Keywords:** Gastroschisis, Neonatal, Predictors, Morbidity, Mortality

## Abstract

Gastroschisis is one of the most common congenital malformations in paediatric surgery. However, there is no consensus regarding the optimal management. The aims of this study were to investigate the management and outcome and to identify predictors of outcome in gastroschisis. A retrospective observational study of neonates with gastroschisis born between 1999 and 2020 was undertaken. Data was extracted from the medical records and Cox regression analysis was used to identify predictors of outcome measured by length of hospital stay (LOS) and duration of parenteral nutrition (PN). In total, 114 patients were included. Caesarean section was performed in 105 (92.1%) at a median gestational age (GA) of 36 weeks (range 29–38) whereof (46) 43.8% were urgent. Primary closure was achieved in 82% of the neonates. Overall survival was 98.2%. One of the deaths was caused by abdominal compartment syndrome and one patient with intestinal failure–associated liver disease died from sepsis. None of the deceased patients was born after 2005. Median time on mechanical ventilation was 22 h. Low GA, staged closure, intestinal atresia, and sepsis were independent predictors of longer LOS and duration on PN. In addition, male sex was an independent predictor of longer LOS.

*Conclusion*: Management of gastroschisis according to our protocol was successful with a high survival rate, no deaths in neonates born after 2005, and favourable results in LOS, duration on PN, and time on mechanical ventilation compared to other reports. Multicentre registry with long-term follow-up is required to establish the best management of gastroschisis.

**Table:** 

**What is Known:** *• Gastroschisis is one of the most common congenital malformations in paediatric surgery with increasing incidence.* *• There is no consensus among clinicians regarding the optimal management of gastroschisis.*	
**What is New:** *• Although primary closure was achieved in 82% of the patients, mortality rate was very low (1.8%) with no deaths in neonates born after 2005 following the introduction of measurement of intraabdominal pressure at closure.* *• Low gestational age, staged closure, intestinal atresia, sepsis, and male sex were independent predictors of longer length of hospital stay.*

**What is Known:**

*• Gastroschisis is one of the most common congenital malformations in paediatric surgery with increasing incidence.*

*• There is no consensus among clinicians regarding the optimal management of gastroschisis.*

**What is New:**

*• Although primary closure was achieved in 82% of the patients, mortality rate was very low (1.8%) with no deaths in neonates born after 2005 following the introduction of measurement of intraabdominal pressure at closure.*

*• Low gestational age, staged closure, intestinal atresia, sepsis, and male sex were independent predictors of longer length of hospital stay.*

## Introduction

Gastroschisis is a congenital abdominal wall defect with herniated intraabdominal viscera exposed to amniotic fluid during pregnancy. The condition is one of the most common birth defects in paediatric surgery with a prevalence of 4.9 per 10,000 live births [1, 2]. Gastroschisis can be divided into two groups, complex and simple gastroschisis. Complex gastroschisis is usually defined by the presence of intestinal atresia, perforation, necrotic segments, or volvulus [3, 4]. Complex gastroschisis is estimated to occur in one-third of pregnancies affected by gastroschisis [3].

Nowadays, survival is more than 90% in neonates with gastroschisis [5–8], yet the condition is associated with significant morbidity [4, 9–13]. Intestinal dysfunction, sepsis, and reoperations result in prolonged duration of hospital stay and parenteral nutrition (PN) [4, 9–13].

In high-income countries, the prenatal diagnosis of gastroschisis is made by ultrasound in more than 90% of the cases [14]. Despite prenatal diagnosis intra-uterine foetal death is still seven-fold higher compared to the general population [15]. Prenatal diagnosis provides an opportunity for clinicians to plan the delivery and perform close foetal surveillance. However, it is difficult to counsel the parents due to lack of consensus among paediatric surgeons regarding optimal timing and route of delivery, choice of surgical technique, and predictors of adverse outcome [15–28]

The aims of this study were to investigate the management and outcome of gastroschisis in a single paediatric surgical centre and to identify predictors of impaired outcome measured by length of hospital stay (LOS) and duration of PN.

## Methods

### Patients

We conducted a retrospective observational study including all neonates with a diagnosis of gastroschisis according to the International Classification of Disease, ICD (Q79.3), who underwent surgical repair at University Children’s Hospital in Uppsala, Sweden, from 1 January 1999 to 31 June 2020. The study was approved by the Regional Ethical Review Board (Dnr 2009/392, Dnr 07/2020). The Uppsala University Children’s Hospital serves a population of 2.5 million inhabitants. The need for patients’ or parents’ written consent was deemed unnecessary by the institutional review boards as we did not contact the families to conduct this retrospective study. Patients were identified in the hospital discharge database by their unique ten-digit birth identification number.

All women expecting a foetus with suspected gastroschisis were referred to our University Hospital and after confirmation of the diagnosis by ultrasound, the delivery by elective caesarean was planned at 36 completed gestational weeks. After delivery, the viscera were covered with warm saline-soaked gauze and plastic or placed in a sterile polyethylene bag (Neohelp™, Vygon (UK) Ltd). A nasogastric tube was placed to decompress the stomach and viscera and to prevent aspiration. The neonate was then transported to the operating room, anaesthetised, and intubated and decompression of the distal bowel was performed by Gastrografin diluted in sterile water (25%) via insertion of a catheter into the rectum. The bowel contents were then gently milked along the bowel to the anus. Primary closure was attempted if the viscera could be replaced into the abdominal cavity without excessive intraabdominal pressure or compromised ventilation. The fascia defect was closed with interrupted sutures and a purse-string type skin closure around the umbilical stump was performed to create a scar with a natural looking umbilicus. If primary closure was impossible, staged closure with the placement of a silobag made by Goretex or a preformed silobag (Bentec Medical) and gradual decompression of the bowel into the abdominal cavity was performed. A Goretex patch was applied in patients for whom most of the viscera could be replaced into the abdominal cavity but the abdominal fascia could not be closed without excessive intraabdominal pressure. Intraabdominal pressure was monitored by measuring intravesical pressure as previously described [29]. The measurement of the intraabdominal pressure by this method has been standard practice since year 2006. This measurement was combined with peak airway pressure, oxygen saturation, and a physical examination by an experienced paediatric surgeon.

All neonates received total PN from the first postoperative day and continued until the establishment of enteral feeding with a stable weight gain. From 2006 to 2009, the PN lipid emulsions were adapted to individually customised PN with a combination of fish oil–based intravenous lipid emulsion (Omegaven®) and an olive oil– and soybean oil–based intravenous lipid emulsion (Clinoleic®) [13]. Minimal enteral feeding with breast milk, either from the mother or from a milk donor, was started after 5 to 7 days, if tolerated. All patients were initially treated with paracetamol and morphine for pain relief.

### Data collection and definitions

Data obtained by review of medical and surgical records was prenatal diagnosis, birth weight, gestational age at birth, mode of delivery, indication for urgent caesarean, sex, maternal age, parity, date and time point for surgery (office hours/on-call hours), duration of intubation, associated anomalies, foetal bowel dilatation, primary or staged closure, technique of staged closure, date at final closure of the abdomen, intraabdominal pressure, reoperations and indications for reoperation, occurrence of necrotising enterocolitis (NEC), episodes of sepsis, intestinal atresia, intestinal necrosis, LOS, days of PN, and survival. Foetal bowel dilatation was defined as bowel dilatation of 10 mm or more. Time point for surgery was defined as office hours (Monday–Friday 8:00–16:30) or as on-call hours. Length of intubation is presented as the total time of intubation during the LOS. Definition of foetal bowel dilatation was 10 mm or more. Time to closure was defined as the time from birth to the time of surgical fascial and skin closure. Sepsis was defined by a positive blood culture in combination with clinical infectious symptoms. The definition of urgent caesarean section was non-scheduled caesarean section. The outcome variables used were survival, LOS, and duration of PN.

### Statistical analyses

Continuous variables were summarised with median (range) and categorical variables were summarised with frequency (%). The comparison between patients with primary and staged closure regarding continuous data was performed using *t*-test or Mann–Whitney *U*-test and for categorical data, chi-2 test and Fisher’s exact test were used.

Duration of PN and LOS were analysed using time-to-event analyses, where the event was defined as discharge from hospital or weaning off PN. Children who died before the event were censored at the time of death. For time-to-event analyses, Kaplan–Meier plots are presented along with the *p*-value for the log rank test comparing the duration on PN and LOS curves. Univariate and multivariate Cox regression analyses were used to predict prolonged LOS and duration of PN. The potential predictors analysed in univariate Cox regression were closure method, foetal bowel dilatation, sex, associated anomalies, intestinal atresia, time point for surgery (office hours/on-call hours), maximum intraabdominal pressure, gestational age, and episodes of sepsis. Variables that had a *p*-value below 0.10 in the univariate analysis were included in the multivariate Cox regression analysis. *p*-values below 0.05 were considered significant. All analyses were performed using R version 3.6.0.

## Results

We identified 114 patients, whereof 52 (45.6%) were females and 62 (54.4%) were males. Characteristics of the patients and their mothers are summarised in Table 1. Most neonates were diagnosed prenatally with gastroschisis (89.5%). Foetal bowel dilatation occurred in 41 patients (48.2%). The majority of the mothers were primiparous (63.2%) with a median maternal age of 24 years. Gestational age (GA) in the neonates ranged from 29 to 38 weeks of gestation with a median GA of 36 weeks. Birth weight was 2515 g (range 1140–3778). The majority of the neonates (105 (92.1%)) were delivered by caesarean section. Out of these 105, 46 (43.8%) were urgent caesarean sections with 24 (52.7%) of the urgent sections dependent on foetal indication.

**Table 1:** Characteristics of neonates with gastroschisis

**Variable**	**Overall**	**Primary closure**	**Staged closure**	**p-value**
*n*	114	94	20	
Prenatal diagnosis, *n* (%)	102 (89.5)	83 (88.3)	19 (95.0)	0.689
Foetal bowel dilatation, *n* (%)	41 (48.2)	32 (45.7)	9 (60.0)	0.471
Maternal age (median [range])	24 [17, 34]	24 [17, 34]	24.5 [20, 34]	0.372
Parity (median [range])	1 [1, 8]	1 [1, 4]	1 [1, 8]	0.253
Caesarean section, *n* (%)	105 (92.1)	86 (91.5)	19 (95.0)	1.000
Urgent caesarean, *n* (%)	46 (43.8)	38 (44.1)	8 (42.1)	0.296
Sex, male, *n* (%)	62 (54.4)	49 (52.1)	13 (65.0)	0.422
Birth weight, g (median [range])	2515 [1140, 3778]	2500 [1140, 3778]	2625 [1787, 3210]	0.773
Gestational age, weeks (median [range])	36 [29, 38]	36 [29, 38]	36 [33, 37]	0.774
Associated anomalies, *n* (%)	4 (3.6)	4 (4.3)	0 (0.0)	1.000
Intestinal atresia, *n* (%)	12 (10.8)	9 (9.7)	3 (16.7)	0.408

Intestinal atresia was found in 12 patients (10.8%). Associated anomalies, other than intestinal atresia, were found in four patients (3.6%). These included comprehensive defects in the brain with dysfunctional tissue in the pituitary gland in combination with dilated ventricles (*n* = 1), Möbius syndrome (*n* = 1), double ureter with the combination of congenital vesicoureteral reflux (*n* = 1), and an anorectal malformation with a vestibular fistula (*n* = 1).

Clinical outcomes after surgery are shown in Table 2. Surgery was performed during office hours in 81 (70.8%) of the patients. Median days until final closure of the abdomen were 7 days (range 2–136). Intraabdominal pressure was measured in 68 (59.6%) of the neonates with the highest measured intraabdominal pressure ranging from 4 to 24 mmHg. NEC was not found in any of our patients. Intestinal necrosis was found in five of the neonates, four at birth, and one after closure of the abdominal wall. Sepsis occurred in 24 of the neonates (21.2%). Reoperation was performed in 13 of the patients (11.5%), where reoperation was necessary once in 10 cases, twice in two cases, and three times in one case. Indications for reoperation were ileus, stricture in an intestinal anastomosis, abdominal compartment syndrome (ACS), and an urachal fistula.

**Table 2:** Clinical outcomes of gastroschisis

Variable	Overall	Primary closure	Staged closure	*p*-value
Surgery during office hours, *n* (%)	81 (70.8)	68 (72.3)	13 (65.0)	0.721
Days until abdominal closure (median [range])	7 [2, 136]	NA [Inf, -Inf]	7 [2, 136]	
Highest intraabdominal pressure, mmHg (median [range])	10 [4, 24]	10 [4, 20]	15 [6, 24]	0.042
Reoperations, *n* (median [range])	0 [0, 3]	0 [0, 3]	0 [0, 1]	0.216
Necrotising enterocolitis, *n* (%)	0 (0.0)	0 (0.0)	0 (0.0)	
Sepsis, *n* (%)	24 (21.2)	18 (19.4)	6 (30.0)	0.365
Necrosis, *n* (%)	5 (4.4)	4 (4.2)	1 (5.0)	1.0
LOS (median [range])	26.5 [2, 199]	25 [9, 199]	46 [2, 183]	0.004
Duration of PN (median [range])	18 [2, 2127]	17 [3, 2127]	36 [2, 628]	0.004
Mortality, *n* (%)	2 (1.8)	1 (1.1)	1 (5.0)	0.321
Time on a ventilator, hours (median [range])	22 [2, 299]	19 [2, 299]	53 [21, 250]	0.000

*LOS*, length of stay; *PN*, parenteral nutrition

Median LOS was 26.5 days and median time on PN 18 days. Median time of mechanical ventilation during hospital stay was 22.0 h. In the study population, 112 of 114 survived (98.2%). One of the deaths was caused by intestinal necrosis due to ACS and one patient with intestinal failure–associated liver disease died from sepsis. None of the deceased patients was born after 2005.

Primary closure was achieved in 94 (82.5%) of the neonates (Table 1). A Goretex patch was applied in four infants where the fascia continuity could not be safely achieved. In one of these infants, the patch was removed due to *Staphylococcus aureus* infection. We found no significant differences between neonates with primary or staged abdominal closure in the characteristics listed in Table 1. Median hours on a ventilator, highest intraabdominal pressure, median LOS, and duration on PN were significantly higher in neonates who underwent staged closure (Table 2).

The method of abdominal closure, sex, occurrence of intestinal atresia, GA, and sepsis were independent predictors of LOS. For every increase in gestational weeks, there was a 1.3 times increased probability of being discharged from hospital at a certain time point. The neonates who underwent staged closure of the abdomen were 60% less likely, those of male sex 42% less likely, those with intestinal atresia 71% less likely, and those with sepsis 63% less likely to be discharged from hospital at a certain time point compared to those without these variables. Foetal bowel dilatation was not a predictor of prolonged LOS.

The independent predictors of duration on PN were the method of abdominal closure, GA, occurrence of intestinal atresia, and sepsis.

**Fig. 1:**
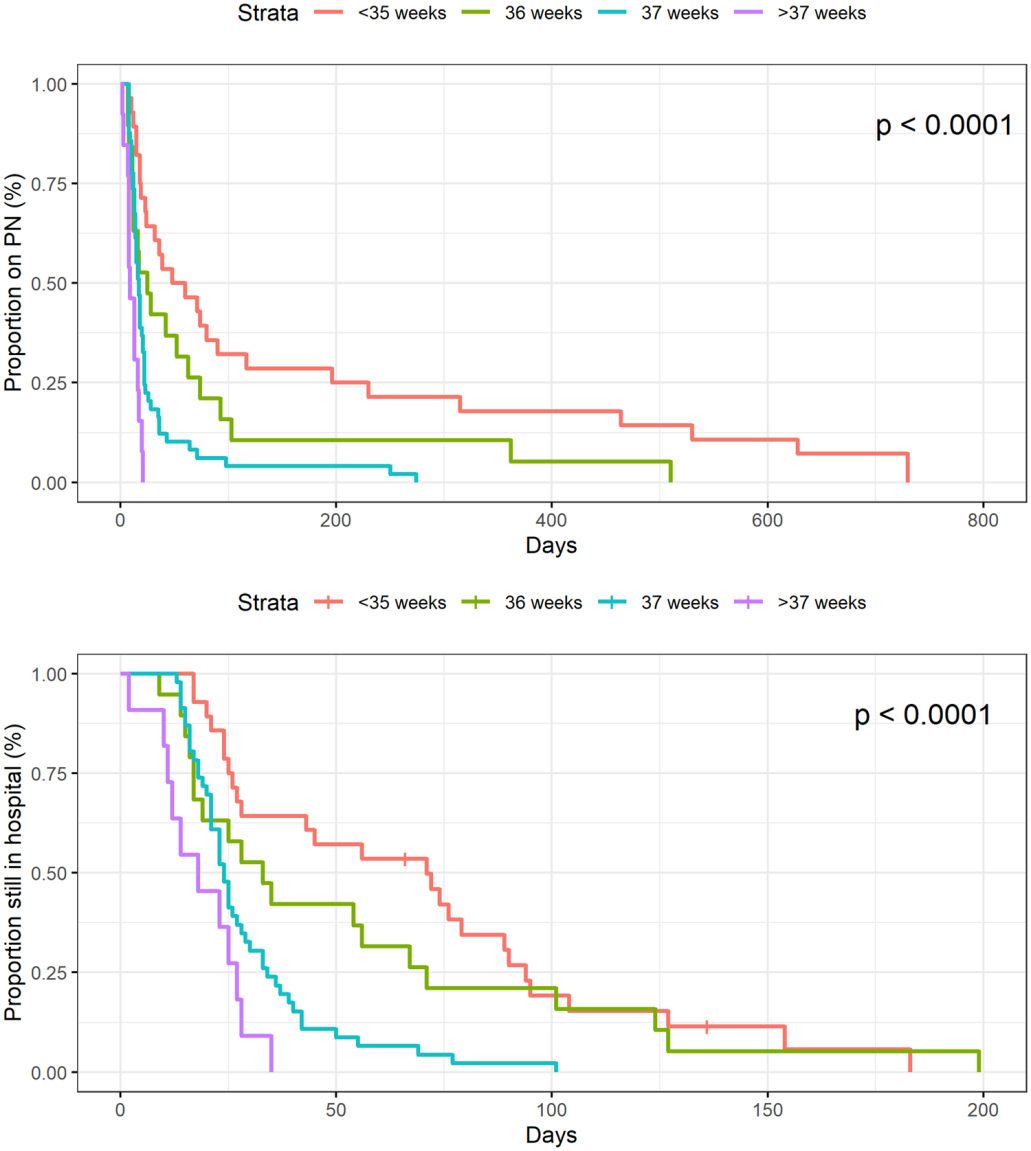
Kaplan–Meier analysis of gastroschisis patients and association between gestational age, duration of parenteral nutrition (PN), and length of hospital stay

The lower the gestational age in the neonate with gastroschisis, the higher the probability of longer LOS and duration on PN as illustrated by the Kaplan–Meier curves in Fig. 1.

## Discussion

In this study, we investigated the outcome in a cohort of neonates with gastroschisis and found an overall high survival rate for hospital discharge (98.2%). The survival rate in our patients was in accordance with other similar studies [7, 30, 31]. A recent multicentre retrospective study from 2020 by Raymond et al. reported a survival rate of 95% in their cohort of 566 neonates with gastroschisis [31].

Since the introduction of individually customised PN with a combination of fish oil–based intravenous lipid emulsion (Omegaven®) and an olive oil– and soybean oil–based intravenous lipid emulsion (Clinoleic®) and the establishment of an intestinal rehabilitation multidisciplinary team, we had no deaths from intestinal failure–associated liver disease or sepsis in our patients with gastroschisis [13]. After we completed our management of gastroschisis with the routine measurement of intraabdominal pressure, no neonates with gastroschisis have died from ACS.

In the present study, median LOS (26.5 days), median duration of PN (18 days), and median time of ventilation (22 h) were favourable compared to those of a recent meta-analysis comprising a total of 1652 patients where LOS was 46.4 ± 5.2 days, duration on PN was 35.3 ± 4.4 days, and length of ventilation was 5.5 ± 2.0 days [8]. A recent national registry study of 849 patients with gastroschisis also found longer LOS (36 days) and PN days (27) compared to our study cohort [32]. Moreover, the publication from 2020 by Raymond et al. reported a longer median LOS (37 days), duration on PN (27 days), and time on mechanical ventilation (5 days) [31]. One explanation for the shorter LOS and time on PN in the present study might be that the neonates could be extubated after in median 22 h compared to other reports in which the neonate was usually on a ventilator for 5 days [7, 8, 16, 31]. The patients could start minimal enteral feeding 5–7 days after surgery. Interestingly, we found that male sex was a predictor of prolonged LOS but not prolonged duration of PN. Prolonged LOS in neonates with intestinal atresia could be explained by prolonged duration of PN due to short bowel syndrome [13].

The practice pattern in the present study was to perform staged closure with silo placement only in patients for whom primary closure failed. Complications from primary closure include ACS and NEC [33]. In the current study, primary closure had a higher success rate (82%) and yet no more complications compared to a study from 2021 in which primary closure was successful in 66% of neonates with gastroschisis [34]. The larger retrospective study by Banyard et al. [33] reported the same rate of primary closure (74%) as a more recent publication by Schmedding et al. [35]. Banyard et al. found that patients undergoing routine silo placement had significantly more ventilator days, longer duration of PN, and longer LOS compared to primary closure [33]. They speculated that the cellular process of bowel healing may not be initiated until abdominal closure has been achieved. We were not surprised to find that staged closure was an independent predictor of prolonged duration of PN and LOS that could be explained by the fact that the intestines in patients undergoing staged closure were in worse condition from the outset [17]. The sepsis rate reported in our study is in line with the rates reported by others [31]. The significantly higher rate of sepsis in the staged closure cohort could be explained by the longer duration on PN since most sepsis episodes were caused by a catheter-related infection. Moreover, longer time to close the abdomen is also a clear risk of contamination and sepsis.

Still there is no consensus on the optimal timing of delivery. Arguments for elective preterm delivery have been reduction of intestinal damage secondary to amniotic fluid exposure as well as the decreased risk of intrauterine foetal death [15]. Intestinal damage may impair absorptive capacity and motility and subsequently prolonged duration of PN. Arguments against elective preterm delivery have been increased mortality, respiratory morbidity, cholestasis, and cognitive defects [36–38]. While some studies have found favourable results with elective preterm delivery [21, 39], others report impaired outcome [19, 22]. We practice elective preterm delivery at 36 completed gestational weeks. The time point of delivery in our neonates with gastroschisis is not contradicted by a Cochrane review and a report from the Canadian Pediatric Surgery Network [40, 41]. They found no significant difference in LOS or in any other neonatal outcomes when preterm birth was planned at 36 weeks, compared with later birth [40, 41]. A systematic review and meta-analysis by Landisch et al. found that elective preterm delivery (< 37 weeks) was associated with a shorter time to first enteral feed and decreased risk of neonatal sepsis compared to those who either delivered spontaneously or had an indicated preterm delivery [20]. The average GA of spontaneous labour in mothers of neonates with gastroschisis is between 36.2 and 36.6 weeks [22, 42]. In the present study, 43.8% of the caesarean sections were urgent caesarean sections. In our study population, around 10% was born after 37 GW and they had shorter duration of PN and LOS. However, elective delivery after 37 GW would lead to increased rate of urgent caesarean sections and the risk of delivery outwith a paediatric surgical centre with complications such as vascular compromise of the intestines due to wrong positioning and delay from birth to surgery that may impair the condition of the bowel. In a previous study, we found longer LOS (median 32 days) when primary closure was performed more than 9 h after birth [5]. The timing of elective delivery in our management of gastroschisis seems appropriate as we are able to perform primary closure in 82% of the study cohort with very low mortality and shorter LOS and duration of PN than in previous studies.

The limitations of this study are the retrospective design and a relatively small study cohort. The strengths are that all the patients during the study period were included and they were treated in the same hospital with a limited number of surgeons.

## Conclusion

The present study shows that the management of gastroschisis according to our protocol was successful with high survival, no deaths in neonates born after 2005, and favourable results in LOS, duration on PN, and time on mechanical ventilation compared to other reports. Low GA, staged closure, sepsis, and intestinal atresia were independent predictors of impaired outcome measured by LOS and duration on PN. In addition, male sex was an independent predictor of LOS. The results from this study could contribute to further knowledge to the management and outcome in gastroschisis. Multicentre registry with long-term follow-up is required to establish the best management of gastroschisis.

## Data Availability

The data and our research material are not available for readers.

## Abbreviations

ACS: Abdominal compartment syndrome
GA: Gestational age
LOS: Length of hospital stay
NEC: Necrotising enterocolitis
PN: Parenteral nutrition
